# Chromosome-level Assembly, Dosage Compensation and Sex-biased Gene Expression in the Small Brown Planthopper, *Laodelphax striatellus*

**DOI:** 10.1093/gbe/evac160

**Published:** 2022-11-01

**Authors:** Qing-Ling Hu, Yu-Xuan Ye, Ji-Chong Zhuo, Hai-Jian Huang, Jun-Min Li, Chuan-Xi Zhang

**Affiliations:** Institute of Insect Science, Zhejiang University, Hangzhou 310058, China; State Key Laboratory for Managing Biotic and Chemical Threats to the Quality and Safety of Agro-Products, Key Laboratory of Biotechnology in Plant Protection of Ministry of Agriculture and Zhejiang Province, Institute of Plant Virology, Ningbo University, Ningbo 315211, China; Institute of Insect Science, Zhejiang University, Hangzhou 310058, China; State Key Laboratory for Managing Biotic and Chemical Threats to the Quality and Safety of Agro-Products, Key Laboratory of Biotechnology in Plant Protection of Ministry of Agriculture and Zhejiang Province, Institute of Plant Virology, Ningbo University, Ningbo 315211, China; State Key Laboratory for Managing Biotic and Chemical Threats to the Quality and Safety of Agro-Products, Key Laboratory of Biotechnology in Plant Protection of Ministry of Agriculture and Zhejiang Province, Institute of Plant Virology, Ningbo University, Ningbo 315211, China; State Key Laboratory for Managing Biotic and Chemical Threats to the Quality and Safety of Agro-Products, Key Laboratory of Biotechnology in Plant Protection of Ministry of Agriculture and Zhejiang Province, Institute of Plant Virology, Ningbo University, Ningbo 315211, China; Institute of Insect Science, Zhejiang University, Hangzhou 310058, China; State Key Laboratory for Managing Biotic and Chemical Threats to the Quality and Safety of Agro-Products, Key Laboratory of Biotechnology in Plant Protection of Ministry of Agriculture and Zhejiang Province, Institute of Plant Virology, Ningbo University, Ningbo 315211, China

**Keywords:** small brown planthopper, chromosome-level assembly, ONT, dosage compensation, sex-biased gene expression

## Abstract

In insects, sex chromosome differentiation often results in unequal gene dosages between sexes. Dosage compensation mechanisms evolve to balance gene expression, but the degree and mechanism of regulation often vary by insect species. In hemipteran species, the small brown planthopper (SBPH), *Laodelphax striatellus*, is an injurious crop pest, with a sex chromosome type XX in females and XO in males. This species offers the opportunity to study dosage compensation and sex-biased gene expression. In this study, we generated a chromosome-level genome of SBPH using Oxford Nanopore Technologies and high-throughput chromatin conformation capture (Hi-C) technology. We also sequenced RNA-seq data from 16 tissue samples to annotate the genome and analyze gene dosage compensation. We finally obtained a 510.2 megabases (Mb) genome with 99.12% of the scaffolds anchored on 15 chromosomes (14 autosomes and 1 X chromosome) and annotated 16,160 protein-coding genes based on full-length cDNA sequencing data. Furthermore, we found complete dosage compensation in all *L. striatellus* somatic tissues, but lack of dosage compensation in gonad tissue testis. We also found that female-biased genes were significantly enriched on the X chromosome in all tissues, whereas male-biased genes in gonad tissues were enriched on autosomes. This study not only provides a high-quality genome assembly but also lays a foundation for a better understanding of the sexual regulatory network in hemipteran insects.

**Significance**

The previous chromosome-level genome of the *Laodelphax striatellus* had relatively low percentage (79.6%) of scaffolds anchored to chromosomes. Moreover, in Hemipteran insects, the dosage compensation level for X chromosomes in different tissues is not well known. We constructed a new genome with 99.13% of the scaffolds anchored on 15 chromosomes. And we found dosage compensation was complete in somatic tissues, while lacking in testis tissue of *L. striatellus*. Third-generation sequencing technology ONT was applied to assemble the draft genome, and Hi-C technology was further applied for chromosomes construction. The transcriptomes from somatic and gonad tissues were used to analyze the dosage compensation extent and sex-biased genes.

## Introduction

Sex chromosome differentiation is a common way to determine sexes in nature (e.g., two basic sex chromosome types: XX females and XY males or ZW females and ZZ males). The sex chromosomes evolve from a normal chromosome pair that carries sex-determining genes (Ohno 1967). The region containing these sex-determining genes will become recombination suppressed, which contributes to subsequent genetic degeneration of sex chromosome regions (e.g., massive gene loss or even complete loss of the Y or W chromosome (Bergero and Charlesworth 2009). This degeneration triggers the problem of unequal gene dosage on sex chromosomes, which could be lethal or deleterious. To mitigate the impact, the mechanism of dosage compensation evolves to balance gene expression on sex chromosomes between females and males and between sex chromosomes and autosomes (Disteche 2012; Graves 2016). Studies involving different lineages have demonstrated that the extent of dosage compensation and the precise molecular mechanisms vary among species. In therian mammals, one entire X chromosome is randomly inactivated in somatic cells of females by epigenetic silencing to balance the dosage difference (Lyon 1961; Gartler and Riggs 1983). In *Drosophila*, dosage compensation is regulated by upregulating the X chromosome in males (Larschan et al. 2011). In birds, reptiles and fish, dosage compensation is partial and gene specific (Ellegren et al. 2007; Vicoso et al. 2013; Shao et al. 2014).

Insects are the most speciose group in the animal kingdom. At present, limited studies regarding dosage compensation have been conducted in certain species of Diptera (Nozawa et al. 2014; Vensko and Stone 2015), Lepidoptera (Catalán et al. 2018; Ranz et al. 2021), Hemiptera (Pal and Vicoso 2015; Richard et al. 2017), Orthoptera (Chauhan et al. 2021), Strepsiptera (Mahajan and Bachtrog 2015), and Coleoptera (Prince et al. 2010), whereas it remains unclear in a tremendous number of other species. Based on existing results, the degree of dosage compensation and regulation mechanisms vary among insect species and even different tissues (Nozawa et al. 2014; Ranz et al. 2021), developmental stages (Rose et al. 2016), and time periods of segment added to X chromosomes (Mahajan and Bachtrog 2015; Gu et al. 2019). Hemiptera is an ideal clade to study the evolution of sex chromosomes. In this group, many species show degenerated Y chromosomes with XY or XO sex determination systems. The dosage compensation in pea aphid, *Acyrthosiphon pisum* (Richard et al. 2017), and three true bugs (Pal and Vicoso 2015) have been analyzed, all show the existence of dosage compensation, but these studies only analyze whole body samples, which could not represent the dosage compensation state among different tissues. In addition, X-chromosome masculinization has been found in pea aphid, whereas feminization has been found in other three true bugs. We still know little about dosage compensation in rice planthoppers. The small brown planthopper, *Laodelphax striatellus* (Fallén) (Delphacidae, Hemiptera), is an important agricultural pest that damages a variety of cereal crops, including rice, wheat, maize, oats, tall oat grass, and barley, as well as transmits several important rice viruses. Its distribution widely covers Asia, Europe and some areas of Oceania and Africa. From 2006 to 2015, rice planthoppers have caused an average of ∼13.1 million tons of economic and yield losses each year in China (Liu et al. 2016).

In recent years, genome assembly combined with RNA-seq data has revealed the state of dosage compensation in many species. The previous draft genome version of SBPH has been assembled by high-depth of Illumina short reads and use low-depth of Pacific Biosciences (PacBio) data to fill the gaps. The scaffold-level genome has a contig N50 of 118 kilobases (kb) and a scaffold N50 of 1.08 Mb (Zhu et al. 2017). Later, this draft genome has been assembled to chromosomal level with Hi-C technology (Ma et al. 2021). However, nearly 20.4% of the genome regions have failed to locate on any chromosome, which may be due to the short length of contig N50 and the lack of restriction sites in many short contigs. The third-generation sequencers represented by PacBio and Oxford Nanopore Technologies (ONT) MinION generate longer reads, reaching tens of kilobases (Laver et al. 2015), greatly improving genome assembly quality. For example, application of ONT and Hi-C data has increased scaffold N50 of Illumina sequence assembly from 356.6 kb to 69.96 Mb for *Nilaparvata lugens* (Ye et al. 2021).

In this study, we assembled the chromosome-level genome of SBPH by combining ONT sequences and Hi-C technology to improve genome quality. By analyzing the transcriptome gene expression, we tested the level of dosage compensation and characterized how sex-biased genes were distributed across chromosomes in different tissues of SBPH.

## Results

### Chromosome-level Assembly of the *L. striatellus* Genome

We sequenced the male genome of *L. striatellus* using four 1D flow cells by ONT 1D. After quality control, a total of 106.89 gigabases (Gb; ∼200X) of clean data was retained, including 16,381,943 subreads with a read N50 length of 21,754 bases and an average read length of 6,525 bases (supplementary table S1, Supplementary Material online). We also sequenced 34 Gb (∼68X) of clean Illumina data of male *L. striatellus* to estimate genome size and polish the genome assembly. The genome length was estimated to be 498,082,955 bp by Genomescope v2.0 with a heterozygosity of 2.9% when kmer was set to 19 (supplementary fig. S1, Supplementary Material online). The subreads were primarily assembled and further polished to obtain the raw contig-level assembly. The raw-contig-level genome was 762,184,311 bp in length, containing 1,731 contigs and the contig N50 was 909,801 bp (supplementary table S2, Supplementary Material online). After removing the haplotigs, a haploid contig-level assembly was obtained in length of 509,864,764 bp, including 603 contigs with a contig N50 of 1,613,830 bp (supplementary table S2, Supplementary Material online).

**Fig. 1.**
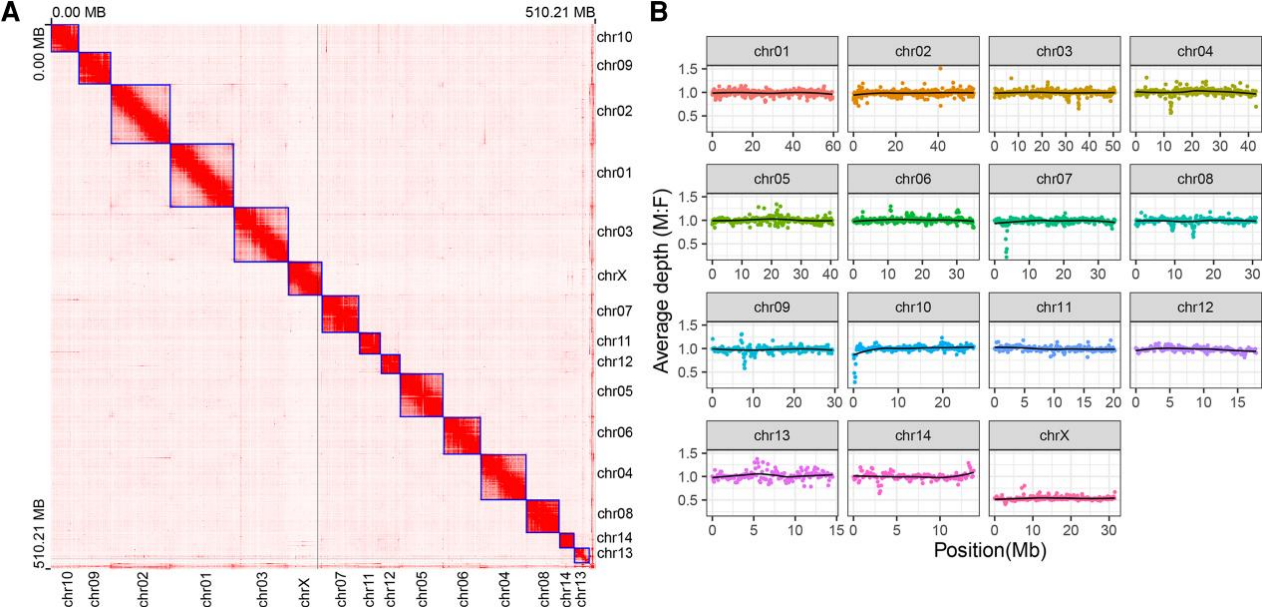
Chromosome genome assembly of *Laodelphax striatellus*. (*A*) Heatmap representing the frequency of Hi-C contacts along the *L. striatellus* genome assembly. Each chromosome was named in order of chromosome length from longest to shortest, as shown on the right and bottom axes. The heatmap was created by Juicebox software using Hi-C data. (*B*) Illumina-sequenced male (M) to female (F) read coverage ratio in a 100-kb fixed window of the *L. striatellus* genome. The lines represent the smooth fitted curve using the LOESS method.

**Fig. 2.**
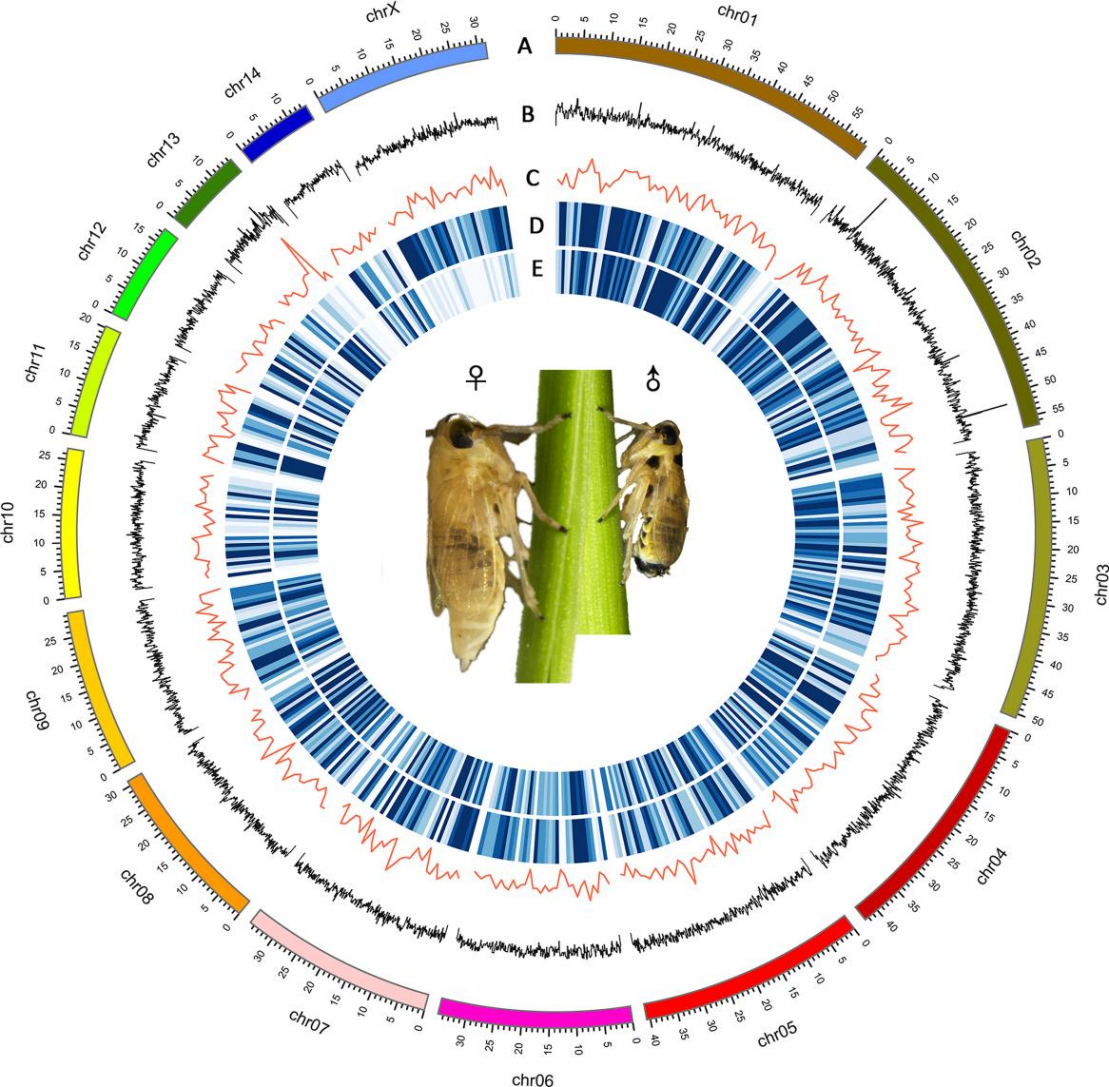
The genome landscape of *Laodelphax striatellus*. (*A*) The ideograms of 15 chromosomes of *L. striatellus*. Black ticks represent genomic intervals of 1 Mb in length. (*B*) Histogram of GC content in a 10-kb fixed window across the genome. (*C*) Gene density lines generated by counting the number of genes in a 1-Mb window across the genome. (*D*) Heatmap showing the number of reads mapped to the genome for transcriptional sequencing data from female ovary tissue. Each block represents a 1-Mb region on the genome. (*E*) Heatmap of transcriptional reads from male testis tissue mapped to the genome in a fixed window of 1 Mb.

**Fig. 3.**
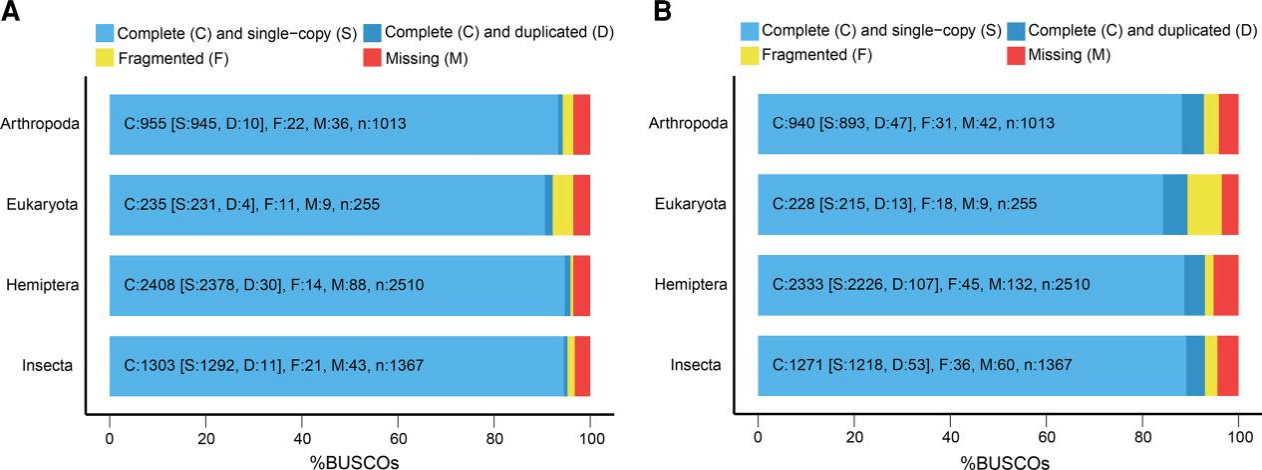
The completeness assessment of the *Laodelphax striatellus* genome using BUSCO. (*A*) The assembled genome was assessed using the Eukaryota (*n* = 255), Arthropoda (*n* = 1,013), Hemiptera (*n* = 2,510), and Insecta (*n* = 1,367) gene sets. (*B*) The completeness of protein-coding genes was assessed using the Eukaryota (*n* = 255), Arthropoda (*n* = 1,013), Hemiptera (*n* = 2,510), and Insecta (*n* = 1,367) gene sets.

We applied Hi-C data (supplementary table S3, Supplementary Material online) to construct haploid contigs for chromosome-level assembly. The length of the final chromosome-level genome was 510,210,580 bp and the scaffold N50 was 34,959,101 bp (table 1). The genome assembly included 15 chromosome-level scaffolds (lengths ranged from 13,928,342 to 59,587,101 bases, accounting for 99.13% of the total genome), and additional 297 small scaffolds (4,460,860 bases in total, accounting for 0.87% of the genome; fig. 1*A*). The GC content of the male *L. striatellus* genome was 34.52% (fig. 2*B*), which was very close to the previous version's 34.54% (Zhu et al. 2017). To assess the completeness of the genome assembly, benchmarking universal single-copy orthologs (BUSCOs) of Eukaryota (*n* = 255), Arthropoda (*n* = 1,013), Hemiptera (*n* = 2,510), and Insecta (*n* = 1,367) were used to align against the genome, and the completeness rate ranged from 92.2% to 95.9% (fig. 3*A*). These results demonstrated the high quality of our genome assembly. Furthermore, we identified a total of 207,334 simple sequence repeats (SSRs) from the whole genome (supplementary table S4, Supplementary Material online). We also characterized a total of 157,801,173 bp of repetitive elements, accounting for 30.93% of the whole genome (supplementary table S5, Supplementary Material online).

**Table 1:** Assembly and Annotation Statistics of Three *Laodelphax striatellus* Genome Version

*L. striatellus* genome version	v1	v2	v3
Sequencing technology	Illumina + PacBio	Hi-C	ONT + Ilumina + Hi-C
Base pairs (Mb)	541	541.1	510.20
% Ns	1.9	2	0.04
Contig numbers	50,020	50,020	1,073
ContigN50 (kb)	71	71	1,362
Scaffolds numbers	20,450	1,908	312
Scaffold N50 (kb)	1,185	29,238	34,959
% of scaffolds anchored in chromosomes	–	79.60%	99.13%
Protein-coding genes	21,254	16,412	16,160
Reference	Zhu et al. (2017)	Ma et al. (2021)	This study

### X Chromosome Identification

The karyotype of *L. striatellus* consists of 15 haploid chromosomes (14A + 1X; Zhu et al. 2017), which is the same number as our Hi-C chromosome-level scaffolds (figs. 1*A* and 2*A*). To identify the X chromosome, ∼34 Gb of males and 33 Gb of females Illumina paired-end DNA short sequences were mapped against the genome to estimate the coverage of the mapped reads. We then calculated male to female coverage ratio in a 100-kb sliding window across chromosomes. The method of sex determination in *L. striatellus* is that females have two X chromosomes (XX) and males have only one X chromosome (XO), so the sequence read coverage of the X chromosome in males should be half that of females. In our results, only one chromosome, the X chromosome, was observed with a male to female coverage of about 0.5, while the ratio of remaining autosomes fluctuated close to 1 (fig. 1*B*).

### Genome Annotation-based on ONT Long-read and Illumina Short-read cDNA Sequencing

To obtain accurate gene structure annotation, we sequenced both Illumina short-read and ONT long-read cDNA data to perform genome annotation. Illumina data were obtained from six independent libraries of *L. striatellus* body tissues, including carcass, fat body, gut, ovary, testis, and salivary glands. After quality control, ∼97.64 Gb of clean data was retained, containing 650,918,608 sequences of 150 bp. Then, these short reads were assembled into 154,426 transcripts by Trinity. ONT data were generated from four independent libraries of *L. striatellus* at different developmental stages, including eggs, nymphs, adult males, and adult females. A total of 8,322,589,622 bases were obtained, consisting of 6,821,102 reads, with an average read length of 1,220 bp. The reads were further classified into 155,475 full-length consensus isoforms with a read length N50 of 3,003 bp (supplementary table S6, Supplementary Material online).

We finally annotated 16,160 protein-coding genes across the genome of *L. striatellus* (supplementary table S7, Supplementary Material online), and 99.34% of genes were anchored on 15 chromosome-level scaffolds. The gene set contained 93% of the conserved Hemiptera (*n* = 2,510) and Insecta (*n* = 1,367) BUSCO genes, respectively (fig. 3*B*). The genes were evenly distributed across the genome (fig. 2*C*). For functional annotation, we aligned 16,160 protein-coding genes against commonly used databases. In summary, 13,399 genes had a hit in the non-redundant protein sequence database (NR), 8,503 genes had a hit in the nucleotide sequence (NT) database, 8,964 genes in the UniProtKB/Swiss-Prot database, 6,907 genes in the Orthologous Groups of proteins (KOG) database, 12,787 in the eggnog-mapper database, 12,369 in the conserved domain database (CDD), and 12,451 in the Interproscan database (supplementary table S8, Supplementary Material online). Furthermore, 10,551 genes were annotated with Gene Ontology (GO) terms (supplementary fig. S2, Supplementary Material online), and 8,192 genes were mapped to Kyoto Encyclopedia of Genes and Genomes (KEGG) pathways (supplementary fig. S3, Supplementary Material online). A total of 14,599 genes were hit at least once in these databases, accounting for 90.3% of the 16,160 annotated genes (supplementary table S8, Supplementary Material online). Overall, the BUSCO completeness and the high annotation rate indicated that our gene annotation was accurate and reliable.

In addition to protein-coding RNAs, we also identified non-coding RNAs (ncRNAs), which are essential components of transcriptional and epigenetic regulation of gene expression. In total, 2,116 ncRNAs were identified across the genome, including 54 microRNAs (miRNAs), 55 ribosomal RNAs (rRNAs), 147 small nuclear RNAs (snRNAs), and 1,860 transfer RNAs (tRNAs; supplementary table S9, Supplementary Material online).

### Dosage Compensation of *L. striatellus*

To investigate whether the degree of dosage compensation differs in different *L. striatellus* body tissues, we obtained Illumina RNA-sequencing data from gonad tissues (female ovaries and male testes) and used publicly available somatic transcriptome data from legs, antennae, and heads (Li et al. 2021). To analyze dosage compensation, we compared gene expression levels between females (XX) and males (X) and between the X chromosome and autosomes within each sex.

**Fig. 4.**
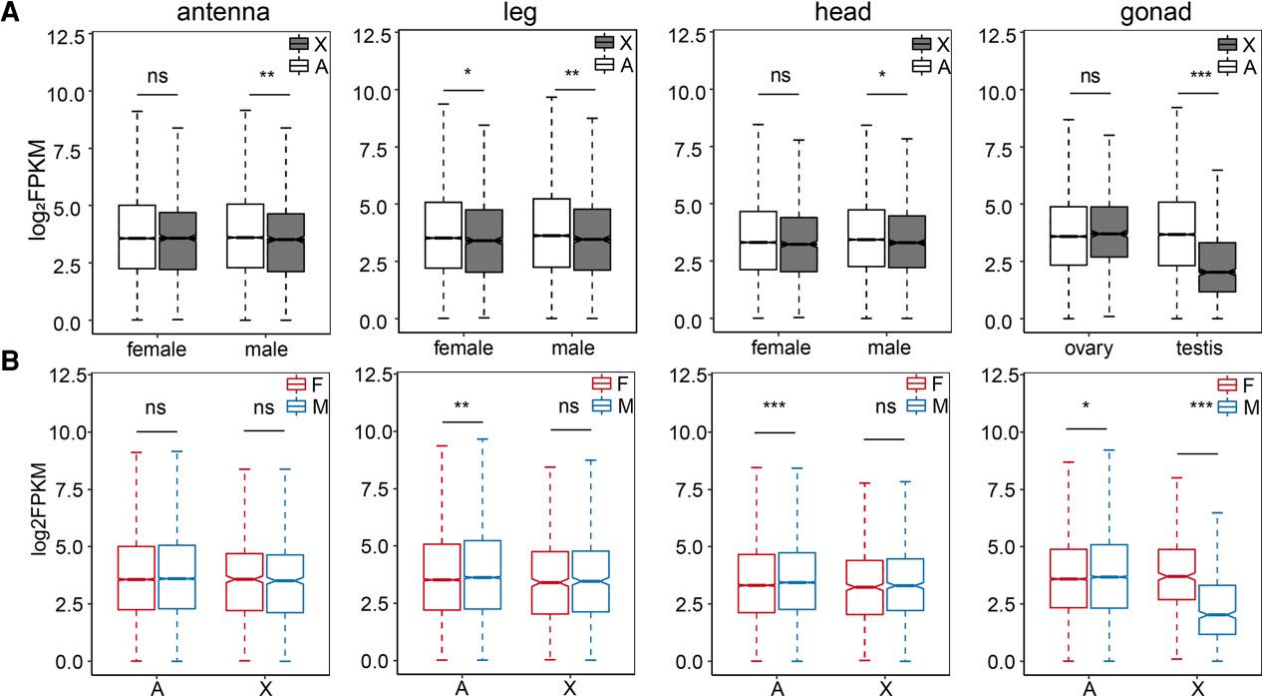
Whole transcriptome expression distribution on chromosomes. (*A*) Gene expression level distribution and significance test between the X chromosome and autosomes within each sex and (*B*) between females and males. Boxplots represent the distribution of normalized gene expression levels log_2_FPKM in females (F) versus males (M) on autosomes (*A*) and X chromosomes (X). The horizontal lines on the top and bottom of the box represent the quartiles, the horizontal line in the middle represents the median, and the two whiskers represent the 1.5*X* interquartile range. Asterisks indicate significant differences between females and males, and significance was determined by Wilcoxon test. *P < 0.05, **P < 0.01, ***P < 0.001, ns, not significant.

**Fig. 5.**
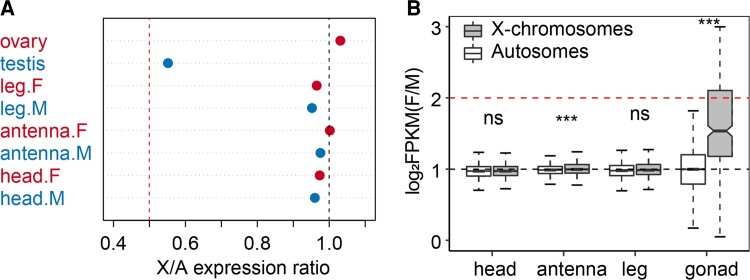
Expression ratios between chromosomes and between sexes. (*A*) Median X-to-autosomal expression ratio (X:A) in females and males. Dots represent the median X-chromosomal expression level log_2_FPKM versus median autosomal expression level (X/A expression ratio) in females (F) and males (M), respectively. (*B*) Boxplots show the distribution of the median female to male expression ratios on autosomes and X chromosomes. The horizontal lines above and below the box represent the quartiles, the horizontal line in the middle represents the median, and the two whiskers represent the 1.5*X* interquartile range. Asterisks indicate significant differences between autosomes and X chromosomes, and significance was determined by Wilcoxon test. ***P < 0.001, ns, not significant.

We first examined the expression levels of the X chromosome versus autosomes in males (X:AA) and females (XX:AA; fig. 4*A*). We found that expression levels of autosomes were significantly higher than X chromosomes in all male tissues (fig. 4*A*; supplementary table S10, Supplementary Material online) (Wilcoxon *P*-values: head, 0.042; antenna, 0.0056; leg, 0.0066; gonad, 2E-16). Testis showed 81%, and somatic tissues showed 3–5% higher median expression in autosomes compared with that of the X chromosome (supplementary table S11, Supplementary Material online). In contrast, there were no significant differences between X chromosomes and autosomes in female tissues (except legs; fig. 4*A*, supplementary table S10, Supplementary Material online; Wilcoxon *P*-values: head, 0.086; antenna, 0.1328; leg, 0.0442; gonad, 0.19). We also investigated the X:A expression ratio of females and males median chromosome expression. We found that in males, the X:A ratio ranged from 0.952 to 0.976, and in females, it ranged from 0.965 to 1.002 in somatic tissues. For gonad tissues, X:A ratio was 0.552 in testis and 1.031 in ovary (fig. 5*A*, supplementary table S10, Supplementary Material online).

In species with dosage compensation, expression should be balanced between one X (X) in males and two X (XX) in females. Therefore, we further compared differences in the X-chromosome expression levels between males and females. We found no significant differences in the X-chromosome expression levels between males and females in all somatic tissues (fig. 4*B*; supplementary table S10, Supplementary Material online; Wilcoxon *P*-values: head, 0.35; antenna, 0.57; leg, 0.86). Furthermore, the median F:M expression ratios for X chromosome ranged from 0.972 to 1.0 in all somatic tissues (fig. 5*B*; supplementary table S10, Supplementary Material online). However, in gonad tissue, we found significantly lower expression levels of X chromosome in male testis than in female ovary (figs. 2*D*,*E* and 4*B*; supplementary table S10, Supplementary Material online; Wilcoxon *P*-value <2E-16). The median F:M expression ratio was 1.76 in the X chromosome (fig. 5*B*; supplementary table S10, Supplementary Material online). When testing for difference in F:M ratio in X chromosomes and autosomes by the Wilcoxon rank-sum test, we found that X chromosomes and autosomes F:M ratios differed significantly in gonad tissue, but not in somatic tissues, with the exception of antenna (fig. 5*B*; Wilcoxon *P*-values: head, 0.7574; antenna, 0.0009; leg, 0.0526; gonad, 2E-16).

Taken together, these results suggest the existence of complete dosage compensation in the somatic tissue of *L. striatellus*. In gonad tissue, however, the male testis lacks dosage compensation.

### Genomic Distribution of sex-biased Genes

**Fig. 6.**
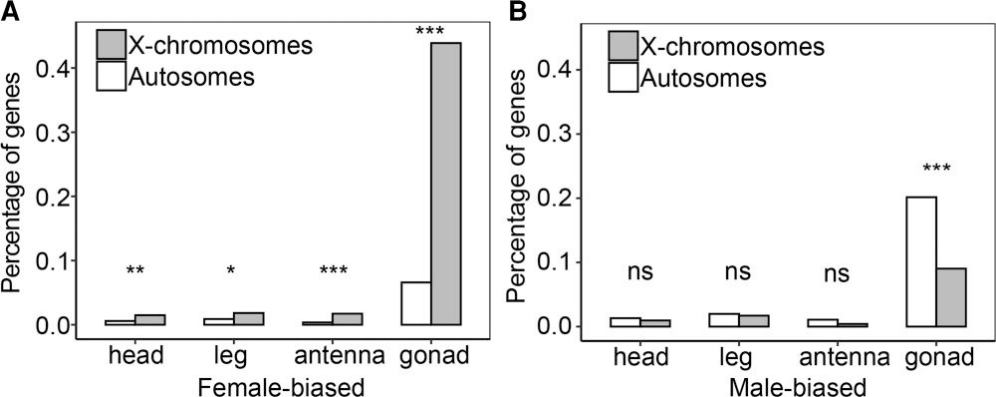
Chromosomal enrichment of female- and male-biased genes. Asterisks indicate significant differences in the proportion of sex-biased genes on autosomes and on X chromosomes. Significance was determined by Fisher's exact test. *P < 0.05, **P < 0.01, ***P < 0.001, ns, not significant. (*A*) Female-biased gene enrichment analysis. (*B*) Male-biased gene enrichment analysis.

Sex-biased genes are often nonrandomly located on chromosomes in many species, with sex chromosomes usually showing a pattern of masculinization or feminization. To analyze whether sex-biased genes have skewed distribution in *L. striatellus*, we used our gene structure annotation results to infer the chromosomal locations of sex-biased and unbiased genes in somatic and gonad tissues. We observed a significantly higher proportion of female-biased genes on X chromosomes than autosomes in all tissues of *L. striatellus*, suggesting an enrichment of female-biased genes on the X chromosome (fig. 6*A*; supplementary table S12, Supplementary Material online). On the other hand, we found a significantly higher proportion of male-biased genes in autosomes compared with X chromosome in gonad tissue. In somatic tissues, the proportion of male-biased genes was higher in autosomes than on the X chromosome, although this difference was not significant (fig. 6*B*; supplementary table S12, Supplementary Material online). Sexual antagonism and possible lack of dosage compensation in the testis may drive X-chromosome enrichment of female-biased genes and autosome enrichment of male-biased genes (see Discussion).

## Discussion

The previous two versions of the *L. striatellus* genome either have a relatively short scaffold N50 (Zhu et al. 2017) or leave ∼20% of scaffolds unplaced on chromosomes (Ma et al. 2021). Here, we improved the genome quality of *L. striatellus*. The new assembly demonstrated higher contiguity than previous versions at both contig and scaffold levels, with contig N50 increased from 71 to 1,362 kb and scaffold N50 increased from 29.24 to 34.96 Mb (table 1). In addition, most scaffolds (99.12%) were anchored to chromosomes, with a lower proportion of ambiguous nucleotides (0.04%). Furthermore, BUSCO analysis showed that 95.9% of the complete Hemiptera orthologous genes were identified in this genome. Thus, the quality of our present *L. striatellus* genome assembly is improved as it is more contiguous, accurate, and complete.

We found that in somatic tissues of *L. striatellus*, the degree of sex chromosome dosage compensation in males was complete. Gu and Walters (2017) argued that sex chromosome dosage compensation should be limited to that heterogametic X-linked gene expression equal to ancestral level of the proto-X, and the equality of sex-linked expression between the sexes was termed as dosage balance. In *L. striatellus*, we found equal level of X chromosome expression between males and females, and no difference in expression between X chromosome and autosomes in homogametic females. Thus, complete compensation with balance existed in *L. striatellus* somatic tissues. Although we found that the X-chromosome expression levels were significantly lower than autosomes (fig. 4*A*), and the median X:A ratios were close to but still <1 in male somatic tissues (fig. 5*A*). First of all, some genes may escape dosage compensation. It has been reported that *Drosophila*'s ability to bear a deficiency in gene dose varied depending on the gene (Lee et al. 2016). Thus, lack of dosage compensation in some genes may cause subtle or even no effect on phenotypes, which may explain the escape of dosage compensation in some genes. In addition, the enrichment of female-biased genes and the absence of male-biased genes on the X chromosome may also contribute to decreased X-chromosome expression in males (fig. 6). Moreover, we also observed significantly higher levels of autosomal expression in males compared with females (fig. 4*B*; supplementary table S10, Supplementary Material online; Wilcoxon *P*-values: head, 0.000017; antenna, 0.18; leg, 0.0061; gonad, 0.02). Therefore, higher autosomal expression may also lead to unbalanced expression of autosomes and X chromosome in males.

For gonad tissue, gene expression levels on the X chromosome were significantly lower in male testes than in female ovaries, reflecting the absence of dosage compensation in male testes. A lack of dosage compensation also has been found in *Drosophila melanogaster* testes, suggesting most of the dosage compensation complex genes are not expressed in the *Drosophila* germline; different testis cells also have different degrees of dosage compensation (Witt et al. 2021). Details of dosage compensation in testis cells and the associated molecular mechanisms require further analysis in *L. striatellus*. Thus, our results show that the degree of dosage compensation differs between somatic and gonad tissues of *L. striatellus*.

Two modes of dosage compensation have been observed in many other species, by downregulating gene expression of two X/Z copies in homogametic sex or upregulating gene expression of one X/Z copy in hemigametic sex. Downregulation of two copies of X results in unequal gene expression between autosomes and X chromosomes in homogametic sex; however, this phenomenon was not observed in female *L. striatellus* (fig. 4*A*). Therefore, the dosage compensation mechanism of *L. striatellus* may be the upregulation of the male X chromosome.

In many species, sex-biased genes often exhibit a nonrandom genomic distribution. We found that the autosomes were rich in male-biased genes, while X chromosomes were rich in female-biased genes (fig. 6*A*and*B*). Similar patterns have been found in *Drosophila* genus, and three possible mechanisms have been proposed to explain these patterns, including dosage compensation, sexual antagonism, and meiotic sex chromosome inactivation (MSCI). Lack of dosage compensation in the *Drosophila* germline is thought to be an important cause of male-biased gene defects in the X chromosome (Bachtrog et al. 2010; Meiklejohn and Presgraves 2012). For *L. striatellus*, a similar scenario is plausible, where the lack of dosage compensation in male testis results in an enrichment of female-biased genes on the X chromosome. However, this mechanism alone does not explain the observations in somatic tissues where female-biased genes were enriched on the X chromosome (fig. 6*A*). In this context, it is possible that another evolutionary force sexual antagonism is at work, namely the opposing selection pressure between females and males may explain the deviations of gene distribution in somatic tissues. In the sexual antagonism theory, dominant mutations with beneficial fitness for females and detrimental fitness for males will be more likely to be fixed on the X chromosome in female homogametic organisms (Rice 1984). MSCI refers to the inactivation of X-linked genes during male meiosis. Since the X chromosome is inactivated in spermatocytes, genes required for sperm maturation are expected to escape to autosomes (Lu and Wu 2011). We observed significantly higher levels of autosomal gene expression in testis than in ovary (fig. 4*B*), and that testis-biased genes were enriched in autosomes (fig. 6*B*). Thus, genes critical for testis development appear to escape to the autosomes, which may indicate the presence of MSCI in SBPH testis; however, the existence of this process in testis remains to be verified.

## Conclusion

This study provided an improved chromosome-level genome assembly of *L. striatellus*. We also confirmed that the degree of dosage compensation varied by tissues in *L. striatellus*. The complete dosage compensation regulation was present in somatic tissues but not in testis tissue. We also characterized the X-chromosome feminization of *L. striatellus*. Lack of dosage compensation in testis, sexual antagonism, and MSCI may contribute to the enrichment of female-biased genes on the X chromosome and an excess of male-biased genes on autosomes.

## Materials and Methods

### Insect Strain and Sample Preparation

The *L. striatellus* strain used in this study was initially collected from rice fields (in Hangzhou, China) in 2018 and reared in the laboratory for >10 generations. The strain was kept at 27 ± 0.5 °C with relative humidity >80% on rice seedlings (Xiushui 134) under a 16-h photoperiod.

### Genome Sequencing

We used the long reads generated by Oxford Nanopore platform to do de novo genome assembly of *L. striatellus*. Genomic DNA from ∼500 male adult was extracted by QIAamp DNA MiniKit (Qiagen) following the manufacturer's instructions. The ONT sequence library was constructed by the ONT 1D Ligation Sequencing Kit (LSK109) according to the manufacturer's procedure and then sequenced on Oxford Nanopore PromethION sequencers. To correct the sequencing error of ONT data, and estimate the genome size, we further sequenced the DNA genomic extracted from 100 unmated male adults on Illumina platform HiSeq X. The Illumina sequencing library was constructed by the NEBNext Ultra II DNA Library Prep Kit.

### Draft Genome Assembly

A genome survey was carried out to estimate the genome size and heterozygosity using the males Illumina paired-end reads by GenomeScope v2.0 (Ranallo-Benavidez et al. 2020), and the kmer was set to 19. The original draft genome was assembled by NextDenovo v.2.4.0 (https://github.com/Nextomics/NextDenovo), the NextCorrect module performed error correction, and the NextGraph module assembled the genome based on the ONT data, and parameters in configuration file were “rerun = 3; read_type = ont; read_cutoff = 1k; genome_size = 500m.” The initial draft genome assembly was further polished twice to correct base errors. The first polish applied the raw Nanopore reads by NextPolish v1.3.1 with default parameters (Hu et al. 2020), and the second polish used Illumina reads by Pilon v1.2.3 with default parameters (Walker et al. 2014). The heterozygosity was reduced by HaploMerger2 with default parameters (Huang et al. 2017) and Purge Haplotigs v1.1.1 was further used to obtain the haploid contigs with parameters “-a 60” (Roach et al. 2018).

### Hi-C Sequencing

Thirty male-fifth instar nymphs were collected for Hi-C library construction. To construct the Hi-C library, the samples were first mechanically disrupted with a homogenizer. The cross-linking reaction was conducted by incubating the cells in 2% formaldehyde. The reaction was stopped by adding 2.5 M glycine. Thereafter, the cells were flash-frozen in liquid nitrogen and stored at −80°C. After cross-linking, the chromatin was digested with 400 IU of MboI restriction enzyme (NEB). The Hi-C library was then prepared by adding biotin, adding T4 DBA ligase (NEB) to ligate DNA, adding proteinase K for reverse cross-linking, DNA fragments purification, fragment shearing to 300–500 bp, and DNA ends repair. Finally, DNA fragments labeled by biotin were separated on Dynabeads® M-280 Streptavidin. The Hi-C library was sequenced on an Illumina Hiseq platform. The Hi-C library construction and sequencing were performed by Novogene Co., Ltd.

### Hi-C Assembly

Chromosomal-level assembly was constructed based on the 3D de novo assembly (3D-DNA) pipeline (Dudchenko et al. 2017). The Hi-C sequencing data were first aligned to the draft genome by Juicer version 1.6 (https://github.com/aidenlab/juicer) to obtain linkage matrix using “-s MboI.” Then 3D-DNA version 180922 (https://github.com/aidenlab/3d-dna) was performed to obtain the chromosomal-level genome using “-r 3.” Juicebox Assembly Tools (JBAT) v1.11.08 (Durand et al. 2018) was used to visualize and modify potential assembly errors before obtaining final high-quality genome. BUSCO version 5.1.3 (Simão et al. 2015) was used to evaluate the completeness of the genome assembly. Basic genomic features including GC content, gene density, and transcriptome reads coverage of ovary and testis were displayed by circos v0.69-8 (Krzywinski et al. 2009).

### X-Chromosome Determination

In the *L. striatellus* sex chromosome determination system, females have two X chromosomes (XX), while males have only one X chromosome (XO). The X chromosome can be identified by calculating the coverage ratio between males and females. DNA sequencing coverage on the X chromosome in males was expected to be approximately half that in females at the same sequencing depth. Therefore, ∼100 unmated males and females were prepared separately for coverage analysis. Genomic DNA extraction and library construction were performed as described above and sequenced on the Illumina Hiseq X platform. Approximately 34G of males and 33G of females clean DNA sequencing reads were generated. The sequencing data were aligned to the genome assembly by BWA v0.7.17-r1188 (Li 2013) with default parameters. The low-quality mapped reads were filtered out by SAMtools v1.10 using “-q 30” (Li et al. 2009). Read coverage was then calculated by sliding a 100-kb window across the genome using the command coverageBed from BEDTools version 2.25.0 (Quinlan and Hall 2010).

### Transcriptome Sequencing

To better annotate the genome, we prepared the transcriptome of *L. striatellus* from representative tissues and developmental stages, and generated both long Nanopore and short Illumina RNA-seq reads. To construct ONT full-length cDNAs, RNA samples were prepared from different developmental stages of *L. striatellus*, including eggs, nymphs, adult females, and adult males. Samples from different body tissues, including carcass, fat body, gut, salivary gland, ovary, and testis, were prepared to sequence on the Illumina platform. All total RNA was extracted by the Takara RNIzol Total RNA Isolation Kit according to the manufacturer's protocols. The ONT and Illumina RNA libraries construction and sequencing were performed by Novogene Co., Ltd following the manufacture's instruction.

### Repetitive Elements and Non-coding RNA Annotation

The repetitive sequences and transposable elements across the genome were identified by RepeatMasker v4.1.2 with default parameters (Chen 2004). Both the eukaryote RepBase library version 20181026 (https://www.girinst.org) and a de novo repeat library constructed by RepeatModeler v2.0.2 with default parameters (Flynn et al. 2020) were used as target databases. SSRs or microsatellites were identified using MISA (Beier et al. 2017). The ncRNAs including miRNAs, snRNAs, and rRNAs were detected using the Rfam database (http://rfam.xfam.org), and tRNAs were predicted by tRNAscan-SE v2.0.9 (Lowe and Eddy 1997).

### Genome Annotation

Protein-coding genes were annotated by combining three lines of evidence, including RNA-seq-based, homology-based, and de novo methods. Illumina short-read cDNA sequences from different tissues of carcass, fat body, gut, salivary gland, ovary, and testis were first assembled into transcripts by Trinity v2.11.0 with default parameters (Haas et al. 2013). ONT reads from eggs, nymphs, adult females, and adult males were mapped to the genome using Minimap2 v2.17-r941 (Li 2018), and rnaSPAdes v3.14.1 (Bushmanova et al. 2019) was used to obtain full-length transcripts with default parameters. Illumina-assembled transcripts and ONT full-length transcripts were mapped to the genome by PASA v2.4.1 with default parameters (Haas et al. 2003) to predict gene structures. GeneWise v2.4.1 (Birney et al. 2004) was applied for homology prediction. Before de novo prediction, repeat sequences were soft masked. AUGUSTUS v3.4.0 (Stanke et al. 2006), SNAP version 20060728 (Korf 2004), GeneMark-ET Suite v4.59 (Lomsadze et al. 2014), BRAKER v2.1.2 (Hoff et al. 2016), and MAKER v3.01.03 (Holt and Yandell 2011) were implemented separately to perform ab initio gene prediction. EVidenceModeler v1.1.1 (EVM; Haas et al. 2008) was used to merge the above results to the final annotation results with parameters “–segmentSize 1000000 –overlapSize 10000” and weights “ab initio, 1; homologs, 5; transcripts, 10.”

Functional annotation of protein-coding genes was performed by aligning sequences to NR (Deng et al. 2006), NT, UniProtKB/Swiss-Prot (Bairoch and Boeckmann 1991), and KOG databases using BLAST (Altschul et al. 1990; *E*-value < 1e-5). Sequences aligned to CDD using the webserver (https://www.ncbi.nlm.nih.gov/Structure/bwrpsb/bwrpsb.cgi). The softwares eggnog-mapper v2.1.5 (Huerta-Cepas et al. 2017) and Interproscan v5.53-87.0 (Jones et al. 2014) were used to obtain the GO annotations. The KEGG pathways were annotated using the KEGG Automatic Annotation Server (Moriya et al. 2007).

### Dosage Compensation

Illumina RNA-seq data for gonad tissues (testis and ovary) were generated as described above, and two biological replicates were prepared for each. RNA-seq data for the somatic tissues leg, antenna, and head were downloaded from publicly available database with no biological replicate (Li et al. 2021). RSEM v1.3.1 (Li and Dewey 2011) was applied to calculate the FPKM expression matrix using default settings, and TMM normalization was applied across all samples using the run_TMM_scale_matrix.pl script in Trinity v2.11.0 (Haas et al. 2013). The relationship between biological replicates of gonad tissues was assessed by Spearman's rank correlation, with correlation coefficients of ∼0.97 for both ovary and testis (supplementary table S13, Supplementary Material online). The average expression of all replicates for each gene was used for the following analysis. We filtered genes with an average expression level of FPKM < 1 in males and females, as this approach has been used in dosage compensation studies for other species (Pal and Vicoso 2015; Catalán et al. 2018). We then log-transformed autosomal and X-chromosomal expression to obtain log_2_FPKM values in males and females. The Wilcoxon rank-sum test was applied to detect bias in female and male expression. The X:A expression ratio was calculated by dividing the median gene expression on the X chromosome by the median of all genes expressed on autosomes. The deviation of the female:male expression ratio on the X chromosome and autosomes was tested by the Wilcoxon rank-sum test.

### Sex-biased Gene Distribution

The RNA-seq data were mapped to the genome using HISAT2 v2.1.0 with default parameters (Kim et al. 2019), and then featureCounts v2.02 (Liao et al. 2014) was applied to calculate the raw read count of each gene using parameters “-p -B -C -t exon -g gene_id.” Sex-biased genes were identified by the Bioconductor package edgeR v3.32.1 (Robinson et al. 2010) as conducted in R v4.0.2 (Team 2013). For gonad tissues with biological replicates, we retained genes with normalized counts per million (CPM) value >1 in both two replications. We then estimated dispersion using the tag-wise method, and applied a generalized log-linear model to fit the one-coefficient analysis. For somatic tissues, due to the downloaded data from public database had no biological replication, we retained genes with CPM normalized value >1. The square-root dispersion (bcv) was set to 0.2. All *P*-values were corrected with Benjamini and Hochberg methods (Benjamini and Hochberg 1995). Sex-biased genes were identified as those genes showing logFC >2 or <−2 and false discovery rate <0.05 (supplementary tables S14–S17, Supplementary Material online). Chromosome counts for sex-biased and unbiased genes on autosomes and X chromosomes formed a 2 × 2 contingency table, and Fisher's exact test was carried out to test the deviation of gene proportions located on X chromosomes and autosomes.

## Supplementary Material

evac160_Supplementary_Data

## Data Availability

The Nanopore and Illumina sequencing data were submitted to the NCBI Sequence Read Archive (SRA) database under BioProject accession number PRJNA841412. This Whole Genome Shotgun project has been deposited at GenBank under the accession JADWYY000000000. The version described in this paper is version JADWYY010000000. The gene structure annotation and gene functional annotation files were deposited at figshare (https://doi.org/10.6084/m9.figshare.20751286.v2).
