# Supplementary material for: Chromosome-level Assembly, Dosage Compensation and Sex-biased Gene Expression in the Small Brown Planthopper, Laodelphax striatellus
Source: Genome Biol Evol. 2022 Nov 1;14(11):evac160. doi: 10.1093/gbe/evac160 (PMC9651030; doi:10.1093/gbe/evac160)
Supplement: evac160_Supplementary_Data [file evac160_supplementary_data.zip › supplmentary_material20221020.pdf]

Supplementary Figures

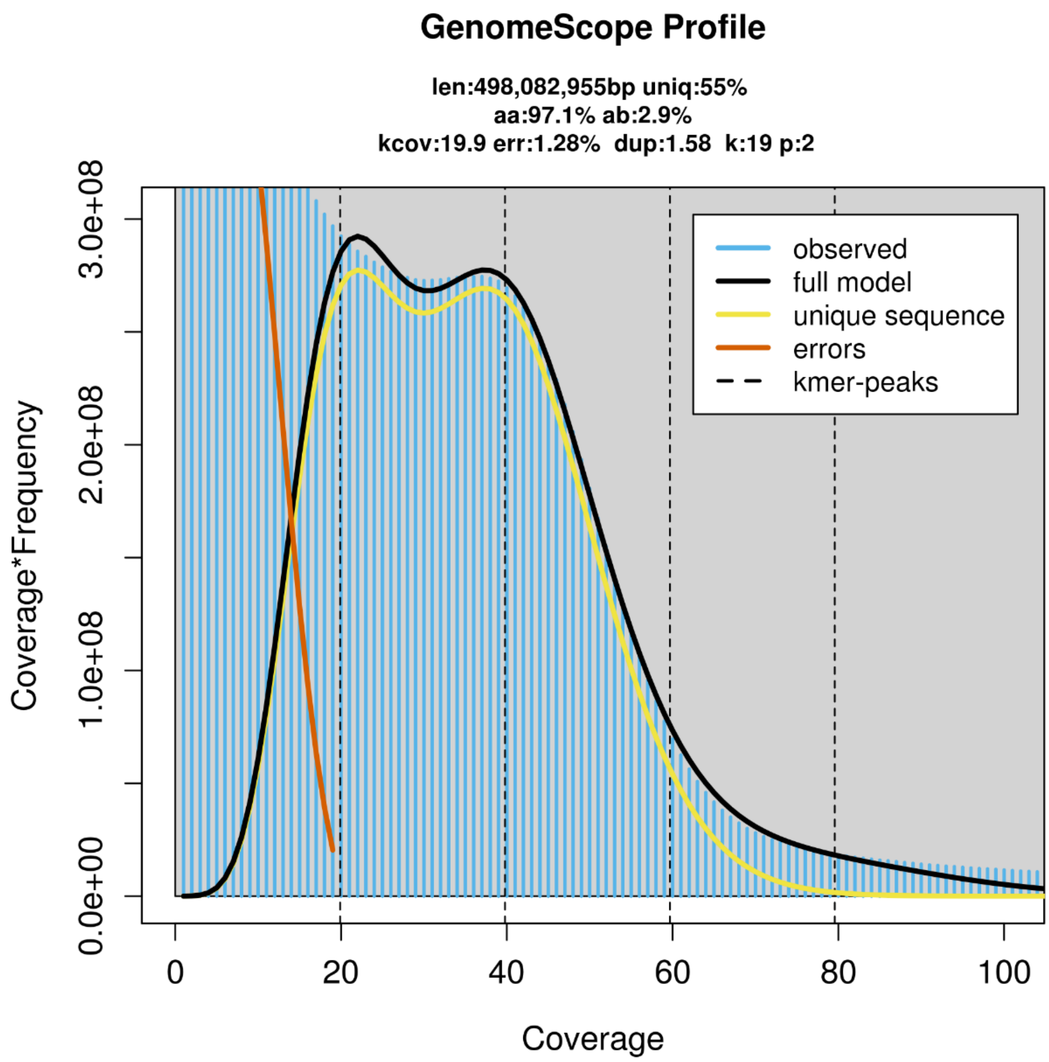

Fig. S1. K-mer distribution based on the Illumina short reads was used to estimate the genome size by GenomeScope v2.0, with kmer set to 19. Homozygous (aa): 97.02%-97.19%; heterozygous (ab): 2.81%-2.98%; genome haploid length: 491,845,871bp-498,082,955bp.

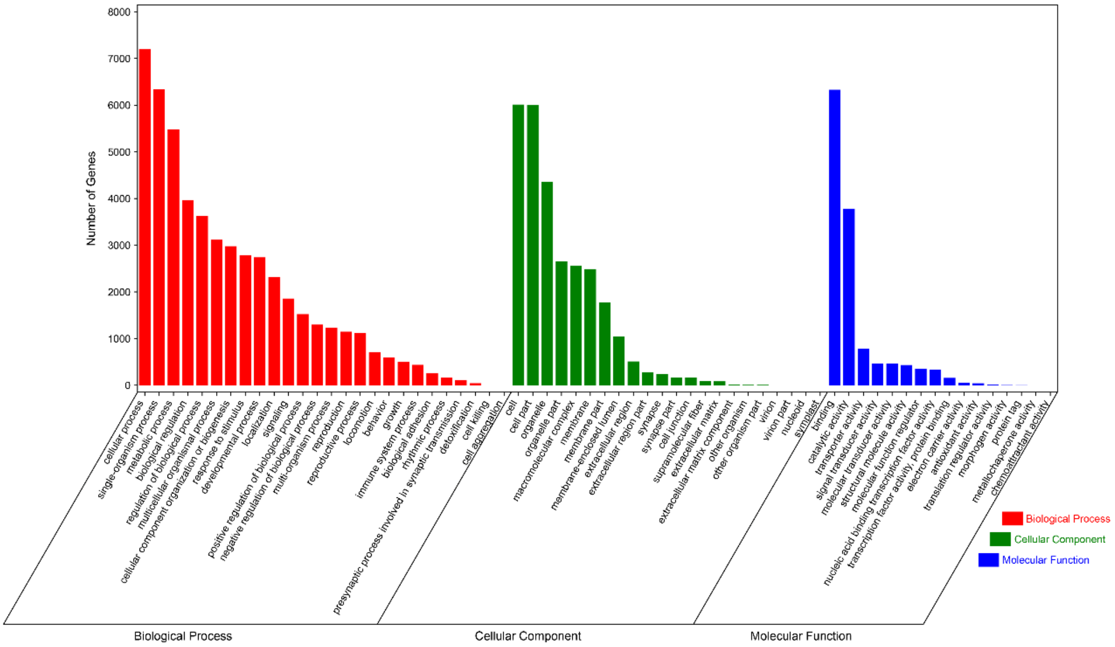

Fig. S2. Gene Ontology enrichment analysis of whole genome genes. The level2 GO terms are shown.

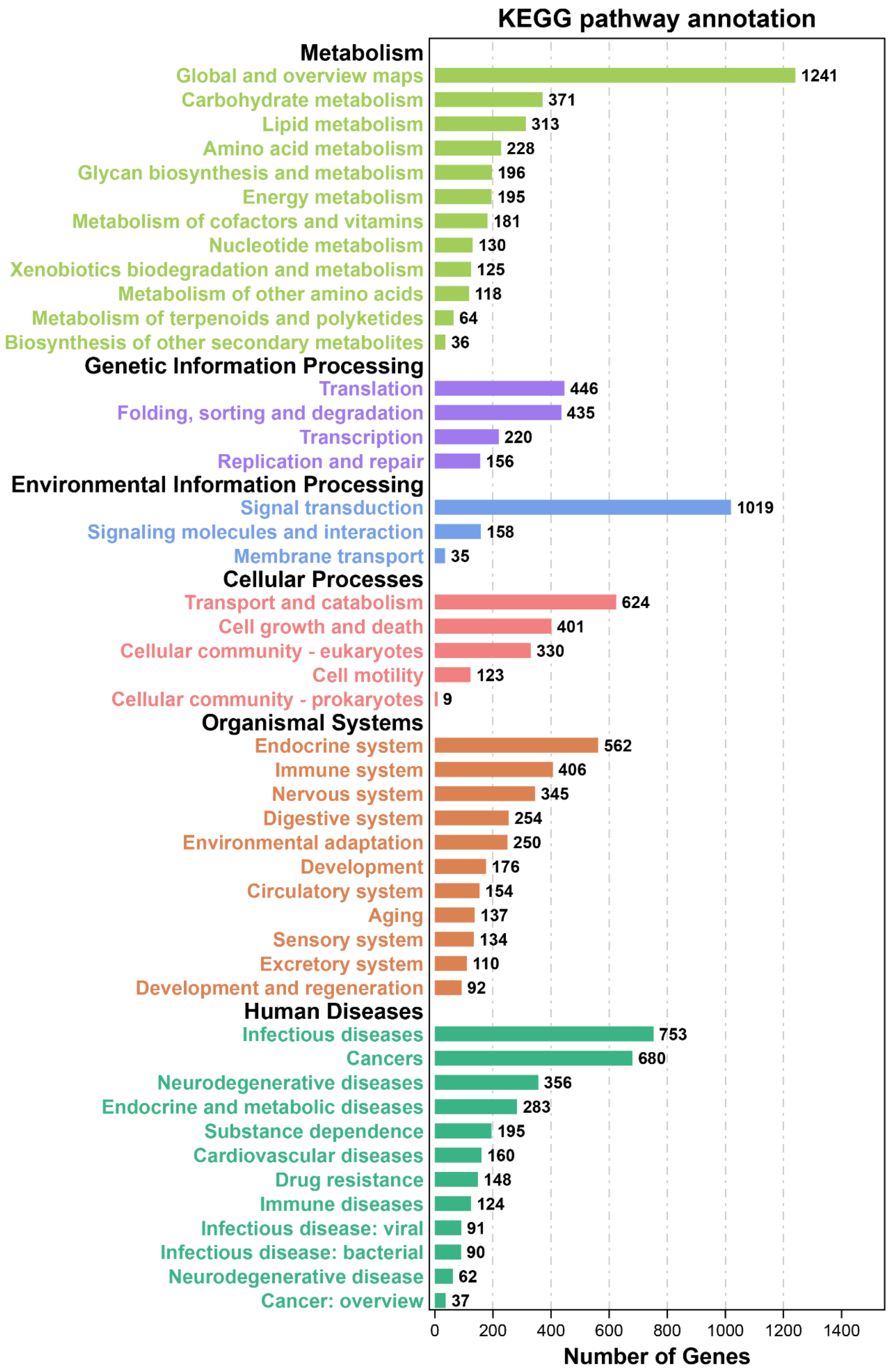

Fig. S3. KEGG pathway annotation for whole genome genes of *Laodelphax striatellus*.
